# An exploratory qualitative study of health professional perspectives on clinical outcomes in UK orthotic practice

**DOI:** 10.1186/s13047-020-00416-w

**Published:** 2020-07-29

**Authors:** Natalie Hall, Daniel Parker, Anita Williams

**Affiliations:** 1 Orthotics Department, East Lancashire Hospitals NHS Trust, Lancashire, UK; 2 School of Health and Society University of Salford, Salford, UK

**Keywords:** Orthotic services, Orthoses, Outcome measures, Qualitative

## Abstract

**Background:**

Despite potential savings to the National Health Service, the collection of data on outcomes of NHS orthotic services is patchy. Indeed, several reports into orthotic services in the UK have reported a lack of data relating to outcomes of care and highlighted the need to routinely measure outcomes to demonstrate efficacy of services. Whilst a previous study provided an overview of the use of outcome measures in orthotic practice and identified some barriers to their use, further questions emerged. Hence, this qualitative study aimed to explore orthotists’ opinions and personal experiences on the influences on outcomes, how appropriate and relevant outcomes can be measured and also how barriers to the use of outcome measures can be overcome.

**Methods:**

Following a review of the literature**,** an initial advisory group informed semi-structured questions. These were used to create dialogue in a focus group of 12 orthotists. Data from the focus group was transcribed verbatim and analysed using thematic analysis, creating themes and subthemes for discussion.

**Results:**

The setting of realistic and agreed goals through managing expectations, compromise and patient education/information were seen as factors that could inform and improve outcomes. Barriers to the collection of outcome measures were associated with inadequate technology to manage the data, lack of time to complete them, lack of training in them and difficulties selecting appropriate outcome measures for patients with complex problems managed by different health professionals. The participants discussed ways of addressing these barriers, such as the use of ‘snapshots’ and delegation of data collection.

**Conclusions:**

This study has revealed that measuring outcomes is considered to be an important activity. In order to achieve good outcomes, it is important to address patient expectations, discuss and establish joint goals for care at the outset and inform and include patients in the decision-making process. The identified barriers to measuring outcomes can be overcome with the solutions revealed by these participants. Hence, this study has contributed to current knowledge which has relevance for clinical practice and may provide the theoretical basis for future research.

## Background

The number of people in the UK with long term conditions such as diabetes, cardiovascular disease, musculoskeletal disorders and obesity is increasing [1] and this is compounded by the fact that by the year 2050, nineteen million people in the UK will be aged over sixty-five and eight million will be aged over eighty [2].

In order to improve the quality of life of people with long term conditions orthoses are described as assisting their mobility and hence independence [3–5]. They also improve quality of life by reducing pain and the need for more invasive and expensive interventions, such as surgery and also social care [5]. It is estimated that for every £1 spent on an orthosis the NHS saves £4 due to the reduced impact on other services [6]. Over half the expenditure for orthotic services is on therapeutic footwear [6] and it is recommended in guidelines [7, 8]. The guidelines are applied despite inconclusive evidence for its effectiveness and evidence that indicates poor levels of use and hence affects the potential for positive outcomes. Also, reports indicate a lack of routinely collecting health outcome data in clinical practice [4–6]. Health outcomes can be defined as the change in health that results directly from an intervention that has been provided. They are important because they provide the ability to understand the benefits and efficacy of orthoses in improving the health of patients and the economic benefits [9, 10].

An NHS mandate [11] required hospitals to publish health outcome data and the use of patient feedback with the purpose of influencing the funding of services. Hence, in order to demonstrate efficacy for the estimated two million orthotic service users and ensure that funding is provided for orthotic services, there is an identified need to routinely audit and collect relevant data on outcomes of care [4–6].

Despite this need there is little evidence on the outcomes of orthotic care in the UK, with less than 30 % of orthotists collecting outcome data for orthotic interventions [12]. It is postulated that this can be a complex task due to the wide range of medical conditions that would require specific outcome measurement tools within orthotic services [13]. Further, there are differing opinions and confusion as to which outcomes of orthotic care are considered important [13] as well as a lack of clinical time and resources available to administer them [12].

Whilst the survey carried out by Young et al. [12] provided an overview of the use of outcome measures in orthotic practice and identified some barriers to their use, questions remained unanswered. Hence, this qualitative study aimed to explore orthotists’ opinions and personal experiences on the influences on outcomes, how appropriate and relevant outcomes can be measured and also how barriers to the use of outcome measures can be overcome.

## Method

### Research approach

An overarching qualitative, interpretivist approach to the research was used. This supports the view that truth and knowledge are established at an individual level and that researchers cannot separate themselves from their own beliefs, in fact these will inevitably influence the whole research process [13, 14]. The researcher (NH) was also an orthotist but carried out the research knowing that an important part of the process would be to regularly challenge and question her own beliefs and interpretations through critical reflexivity in order to demonstrate credibility [15]. Ethical approval (University of Salford (UoS) (HSR1617–113) and IRAS (17/NW/0379).

### Participants

Purposive sampling was undertaken in order to select those who met the inclusion criteria. The inclusion criteria were those currently working in a professional role within orthotic services, an interest in outcomes and measurement of outcomes and agreeing to attend a focus group. The chair of the group was initially approached in relation to recruitment and also arranging a date for the focus group to take place. Forty-three members of the NHS Orthotic Managers Group (NOMAG) were invited to participate by email invitation. These potential participants were provided with participant information about the study. The focus group was conducted at the groups scheduled meeting where 15 attended and all agreed to take part.

### Data collection

A face to face focus group was the method chosen for data collection in order to share opinions and create discussion between participants allowing the researcher (NH) to gather rich and insightful data [16]. It took place at the Birmingham Exhibition Centre in a meeting room on 19/10/17.

The questions for the topic guide were developed from a review of the literature on outcomes and measurement of outcomes and aimed to direct the discussion but not to set it. Hence the questions were not piloted. These were refined with input from an advisory group consisting of two orthopaedic consultants, a senior physiotherapist, senior podiatrist and a senior orthotist.

Informed consent was obtained before commencement of the focus group. Demographic information was collected in the form of a questionnaire just prior to the start of the focus group. The researcher used a focus group guide to ensure that participants understood the aims of the study and the process of the focus group. The focus group was digitally recorded, and field notes were taken by a member of NOMAG in order to demonstrate confirmability. The focus group lasted for 70 mins.

### Data analysis

The researcher transcribed the audio recordings verbatim and analysed alongside observations recorded in the field notes. Data was organised manually. Each participant was allocated a participant number in order to maintain anonymity and confidentiality.

An inductive approach to thematic analysis of the data was undertaken [17]. The researcher familiarised herself with the data then created initial codes and categories. This informed the search for themes which were then reviewed, defined and named with agreement from co-authors and then advisory group. Appropriate quotes were extracted from the data and used to illuminate the themes. The interpretations of data were shared with the co-authors and advisory group for confirmability.

## Results

Fifteen participants took part in the study (Table 1) and were all currently employed in NHS orthotic services in England. Those in management positions had clinical experience, and all stated that most of their practice focused on footwear. Eighty percent [*n* = 12] of the participants stated that the measurement of outcomes was relevant to practice but only 40% [*n* = 6] used them in practice.

**Table 1:** Participant Demographics

Participant Number	Gender (M/F)	Current Role	Years In current Role
P1	M	Head of Service/ Orthotist.	15
P2	F	Head of Service/ Clinical Lead Orthotist.	3
P3	F	Orthotic Service Manager.	7
P4	F	Clinical Lead Orthotist.	1
P5	M	Clinical Service Lead.	9
P6	M	Clinical Lead Orthotist.	15
P7	M	Clinical Lead Orthotist.	No response
P8	M	Senior Orthotist.	No response
P9	M	Clinical Lead/Head of Regional Orthotic Services	1
P10	F	Principal Orthotist.	8
P11	F	Senior Orthotist.	3
P12	F	Clinical Lead Orthotist	21
P13	F	Senior Manager for NHS Orthotics and Wheelchair Services	2
P14	M	Clinical Lead/Orthotist	1
P15	F	Manager of Podiatry and Orthotics	8

### Themes and subthemes

The researcher identified subthemes which informed the subsequent themes (Table 2).

**Table 2:** Themes from the Focus Group

Themes	Title of Theme	Subthemes
**Theme 1**	The Role of Goal Setting.	Conflicting goals. Achieving agreement. Managing expectations / compromise.
**Theme 2**	Achieving Behavioural Change.	Users motivation. The role of education. Acceptance and satisfaction.
**Theme 3**	The Barriers to Measuring Outcomes.	Fluctuating disease and changing goals. Impact of multiple interventions. Time constraints. Varied case load.
**Theme 4**	Overcoming the Barriers to Using Outcome Measures.	What We Need. Role of Technology. Delegation. Snapshots.

### Theme 1: The role of goal setting

#### Subtheme: conflicting goals

Orthotic devices will usually be prescribed with intended goals. Participants talked of the difficulties that arise when other professionals and service users present with their own thoughts and ideas around what an orthosis can achieve:

*“What's the outcome from the initial referrer because it could be totally different from what ours is.”* P6

*“Because what other clinicians feel an orthosis does and what we are telling them it actually does are sometimes very different … You've got the three different concerns you've got the referrers you've got the patients and then you've got ours.”* P1

These differing goals between parties were also discussed when considering the challenges of using outcome measures:

*“We have been using the GAS lite system which relies on you getting what the patient wants, what the orthotist wants and combining the two … the problem is when we actually started doing that, the objectives the patient wanted and what the orthotist wanted weren’t even vaguely close and the perception coming through with the referral and with the referrer didn’t match up with either of them either.”* P7

Participants attributed these differing goals, particularly for patients, to differing priorities. They felt the important goals for clinicians often were not the important goal for patients:

*“ … we are all guilty about still pursuing to make them safer on their feet … make sure we are reducing the level of risk but actually at the end of the day it’s still not the patient’s goal.”* P7

Participants talked of identifying goals at the outset of treatment as an important start towards the measurement of outcomes. However, the different opinions (themselves, the referrer and the patient) on what the intended goal should be is vital in maximising the potential for a positive outcome.

#### Subtheme: achieving agreement

These differing goals meant there was a need for discussion with patients. One participant described the need to establish a joint goal prior to starting orthotic treatment:

*“We see the patients … they’ve got their own ideas, they talk to friends, their families … it’s getting the marriage of those two things.”* P6

The other participants confirmed this, describing agreement of goals with patients as key to successful outcomes:

*“well the question is … can you achieve an outcome that the patient understands what’s wrong with them … why the clinician is suggesting what their suggesting and you’ve agreed on what the goal will be.”* P5

*“It has to meet the patients and the healthcare professional’s expectations of what it aims to do.”* P2

Participants described the need for a process of joint decision making for goals that resulted in an agreed treatment plan.

#### Subtheme: managing expectations/compromise

Linked to goal setting, participants described a need to establish and identify patient expectations prior to treatment:

*“Is the patient’s objective to reduce pain, prevent falls, walk better when you know it is managing those expectations.”* P13

They indicated the need to compromise and readjust the patient’s expectations:

*“The whole point of an orthosis is to achieve a successful clinical function, but if we have actually provided them with something and they are happy … is that really the ultimate goal, even though it may not be as functionally or as clinically appropriate?”* P1

*“You’re going to make something that’s not going to be fantastic but that will fit in the shoes that they wear and will be usable.”* P5

In instances where expectations were considered impossible to meet, questions were posed as to whether treatment was appropriate at all:

*“Because if you establish at the front end that actually the two [goals] won’t ever meet and we’re not going to satisfy the patients expectations why order the product and pursue any further.”* P1

Participants expressed the need for some level of compromise when addressing patient expectations.

### Theme 2: Achieving behavioural change

#### Subtheme: users motivation

Successful outcomes were described as an orthosis being “usable” with motivation being a huge factor in engagement and hence a successful outcome,

*“If you have got someone that’s determined enough to get back to sport then they will wear that massive knee brace, because it means they can go play football again.”* P2

As part of this participants acknowledged the difficulties patients faced in order to change behaviour to use an orthosis:

*“I work a lot with neuro patients and I can see they tip or fall or have a foot drop, and I’ll say well we’ll put an intervention in and that stopped you tripping and you’re not going to fall, but to that patient them falling isn’t a big enough problem for them to change their footwear”* P2

Participants also felt that the cosmetic appearance may detract from the clinical goals,

“ … that’s still the clinical measure and your expectations of what you want for that patient … reduce the Falls … but actually at the end of the day it’s still not the patient’s goal and cosmesis is the big one for that especially if it’s about clothing choices”. P1

#### Subtheme: the role of education

Participants described their role of empowering patients with information to understand the recommendations being made in order to make decisions on their care:

“*… the most powerful tool we have is the ability … to educate our patients … help them understand what’s going on and what your aims are and therefore why you’re going to design something in the way you’re going to design it …*” P5

*“My patients are all very happy but some of them don’t achieve the goal they wanted to achieve, but they’re still very happy because they understood what we were trying to achieve … they felt a part of the process.”* P3

Preparing patients for their potential orthotic treatment could involve education before referral for example:

*“We have an early intervention rehab team … they will refer into my orthotic clinic and part of that is … they are educated … they are more likely to have successful outcomes … they know what to expect, they know what their options are.”* P1

A process of educating patients was viewed as a way of making them feel involved in their care and in the context of their own needs, expectations and personal goals. Although this may not result in achieving maximum health outcomes, it was seen as an important factor in patient engagement and use of the orthoses.

#### Subtheme: acceptance and satisfaction

Participants described the importance of the patient’s eventual acceptance and use of a device as an important outcome:

*“Something that the patient will wear and use, if it goes into a cupboard it’s a useless orthosis no matter how good it is.”* P7

This focus on patient satisfaction and a “usable” device also appeared to influence the types of outcome measure that these participants would use in their practice. However, they expressed a preference for measures of satisfaction:

*“Patient satisfaction from start to finish with their journey and that for me...that’s the one I go to because my patients are happy if they’ve enjoyed their journey … they’ve had a good outcome. I’m happy.”* P5

*“...we looked into outcomes but it’s very much a case of we still use the review or the return of the patient coming back to then think have we achieved it.”* P1

A patients’ use of the orthosis and being satisfied with their care were seen as important outcomes of treatment. Because of this, participants agreed that the use of outcome measures that included satisfaction would be preferable.

### Theme 3: The barriers to measuring outcomes

#### Subtheme: fluctuating disease and changing goals

Participants indicated the pattern of different chronic diseases as the most challenging factor in the measurement of outcomes:

*“What can happen especially with chronic disease is those goals change.”* P6

“*… some of them are chronic, like RA condition … you know on that day might be ok but then after, or an hour after it might not, so it’s really tricky.”* 13

Participants also described how patients themselves could change the goals set at initial assessment and highlighted how patients themselves change goals, with the introduction of new hobbies or new expectations of care during treatment:

“*… a lot of our patients are just long-term chronic patients and their needs change for all sorts of reasons, which can be vocational as well as their disease progression, you know they can change because they decided to take up bowls which is really good for them socially, so we actually need to adapt to that.”* P2

#### Subtheme: impact of multiple interventions

Multidisciplinary management of patients with chronic diseases was seen as a challenge. This will often involve several different treatment modalities and a variety of different health professionals:

*“I think those that are diabetic change things considerably really … there are so many other factors that will determine that outcome it’s very hard to, I think, pin down and hard to measure success based on other variables that you don’t have control over, compliance, diabetic cont*r*ol, vascular supply …*” P5

*“And on the back of that it’s a case of a lot of orthosis, especially for diabetic care are about maintenance and prevention and its quite hard to measure whether it’s successful other than they haven’t re-ulcerated. But they might not have re-ulcerated for many other factors.”* P1

It is clear that orthotic management for patients with chronic disease was rarely provided in isolation and so potential benefits could not always be attributed to orthoses alone. This creates a problem when selecting a specific outcome measure.

#### Subtheme: time constraints

Participants described their experience of using outcome measurement tools in practice as difficult due to the length of time to complete them:

*“I use … the Manchester foot pain disability index; don’t use it routinely as it takes too long.”* P5

*“I’ve used OPUS before … this was the worst outcome measure because of the length of i.t”* P9

Other participants noted the same issues when discussing some outcome measures:

*“The only problem with both of these, they are massive to do …*” P7

*“They are too lengthy to feel like they are practical within a 20/30 minutes appointment slot”* Participant 1

Participants indicated that these outcome measures were not practical for use in their everyday orthotic practice and setting. They indicated the average time slot provided for assessments did not allow for the extra task. They felt a number of outcome measures were too lengthy and indicated that patients would find this an inhibiting factor.

#### Subtheme: varied case load

Participants also described difficulties associated with identifying the most appropriate outcome measures from the vast number available for the various pathologies they treated:

*“There’s a certain amount of apathy with me in the fact that I’m doing a lot of other things … and so when that patient comes through the door who fits the bill for the outcome measure … I’ve ran out of time.”* P1

*“Because within a day, the day starts, and you know you can have your rheumatoid one moment and something else the next, you can’t have all the questionnaires at reception for ‘what type of patient are you?”* P5

*“That’s what’s often the problem isn’t it. It’s a different outcome for everybody.”* P2

The diversity of patient groups made it difficult for participants to identify the right outcome measure at the right time and this was a barrier to routine use.

### Theme 4: Overcoming the barriers to using outcome measures

#### Sub theme – what we need

The participants expressed a preference or need for outcome measures that were quick and easy to use:

*“We need a scale that allows us to have three questions; GAS lite gives us that straight away.”* P7

*“The most effective thing in the vast majority of the population is a really simple text message with three questions.”* P7

Participants favoured the idea of generic measures, applicable for the majority of service users:

*“When we looked at them there was such a huge variety of different outcome measures and we couldn’t find one that gave us a clinical outcome that we could put across the board.”* P10

This appeared to link to the overall preference for patient satisfaction questionnaires:

*“So that’s why it’s always good to have the service one. How were reception staff? Did you feel like you were listened to? Where you given enough time? Did you feel like all your questions were answered? Did you get something that was usable? Do you find that it helps you? Are you happy with the service?”* P5

Participants wanted measures that were manageable to use within the time frames they were given. This appeared to lead to a preference for those that are simple and quick to complete and could incorporate technology for ease of collection. This is why a number of validated outcome measures were not being used by these participants, with a preference towards satisfaction questionnaires completed by patients themselves.

#### Subtheme: role of technology

The traditional paper format of outcome measures was seen as a barrier to their use:

*“the problem is you’ve got all these outcome measures that are stuck in these cupboards and written on Post-it notes, in files, it’s all just lost.”* P9

Technology was suggested as a method in aiding selection:

*“It’s all paper again. We’re miles off technology, you could have all this stuff on an iPad, it’s very easy, you could email it to patients.”* P9

However, participants talked of their frustrations related their current IT systems that did not support this:

*“The IT systems make or break a lot of this as far as measuring because … can’t retrieve as a report … our IT system doesn’t do it.”* P1

Technology was seen as a way of addressing not only the data burden but also the selection and administration of appropriate outcome measures, but the current IT systems did not facilitate it.

#### Subtheme: delegation

To tackle issues related to time constraints participants talked of delegation of measuring outcomes to others:

*“We’re using telephone reviews … telephone reviews are being done by our orthotic assistant.”* P11

*“I also am exploring whether it has to be an orthotist that makes that phone call, whether someone else can make that phone call to ask some risk based questions which would then either send the patient back through triage and for review or we would say that’s a happy person and we have achieved what we wanted.”* P13

Lack of clinical time meant that some participants had been driven to find alternative ways of collecting outcome data.

#### Subtheme: snapshots

Another way in which barriers were addressed was the use of what one participant described as ‘snapshots’:

*“I use … the Manchester foot pain disability index … we will do a full week or a snapshot … it’s a bit labour intensive for that week but it’s only that week.”* P5

*“So, what we decided to do is to have 6 months on an area of a group of patients with one outcome measure for that and then to move on to another cohort of patients and probably with a different outcome measure.”* P10

There was agreement between participants that this was a way of overcoming the barriers:

*“I think … if you did some random sampling maybe in a quota and ran 20, 30, 40, 50 patients, whatever the sample size … you could do a modelling thing.”* P13

Participants agreed on the importance of outcome measures for orthotic practice and looked at novel ways in achieving its collection through ‘snapshots’, focussing on specific patient groups or pathologies over a short time meaning that data collection was more manageable.

## Discussion

This qualitative study has revealed, for the first time, orthotists’ opinions and personal experiences on the influences on outcomes, how appropriate and relevant outcomes can be measured and also how barriers to the use of outcome measures can be overcome. Indeed, goal setting and achieving changes in the patient behaviour were viewed as key to achieving positive outcomes. Whilst the participants identified several barriers to the use of outcome measures, they also shared their opinions and experiences on how to overcome these barriers.

### Role of goal setting

The participants described how they felt successful outcomes in orthotic care hinged on being able to establish agreed goals between the orthotist, the referrer and the patient.

They considered that the assessment process is an opportunity to develop a relationship with patients in order to create individualised goals and identify any potential barriers they could have in achieving them [18]. Further, they indicated that establishing and agreeing patient expectations prior to treatment was key to achieving successful outcomes. Entwistle et al. [19] described how true patient autonomy can often mean a process of establishing new options and expectations. Further, it has been demonstrated that patients who feel involved in their care demonstrate better outcomes [20]. However, shared decision making is not an easy task [21], it involves detailed discussions with patients to establish their expectations and goals. As patients have expectations about the care they will receive and that expectations have been shown to directly influence the outcomes of care in other areas of health care [22–24] there may be a need to readjust expectations if their desired goals are not achievable. However, in the context of orthotic services it is unclear what patient expectations are in relation to outcomes or outcome measurement and this needs investigation. In clinical practice, orthotists should elicit what patients’ expectations are in order to achieve realistic goals and therefore maximise the potential for positive outcomes.

### Achieving behaviour change

Effective communication is crucial to goal setting and shared decision-making and is also considered an important part of patient engagement and satisfaction with care [25]. Employing effective communication should be viewed as an important step in orthotic treatment planning process and in achieving acceptance [26]. Patient information and education has been long advocated as a way to improve health outcomes, with the idea that if a service user understands why recommendations have been made, they are more likely to follow them [27, 28]. Yet this does not mean it is an easy task. Johnson et al. [29] found that health professionals would often make recommendations in relation to footwear which they found too difficult to follow.

It was considered by the participants that if a patient did not utilise their orthoses then inevitably that was an unsuccessful outcome. Therefore, successful outcomes are influenced by the patient’s motivation to accept and implement required behavioural change to use the orthoses [26, 29]. The participants touched on the impact of orthoses on clothing choice, hence non-compliance affecting outcome, and this aligns to a report where up to 90 % of those prescribed footwear were concerned about the cosmetic appearance and found that they had to change their clothing to accommodate or disguise them [30]. Hence, it is vital for orthotists to discuss the impact of the orthoses and in particular therapeutic footwear in relation to cosmesis in order to establish compromise and agreement. This could be key in influencing the eventual use of the orthoses and thus the eventual outcomes of care. Orthotists should consider the ISO definition of usability in relation to goal setting which is, … the extent to which a product can be used by specific users to achieve goals with effectiveness, efficiency, and satisfaction in a specified context of use’ [31].

Janninck et al. [32] applied the concept of ‘usability’ to therapeutic footwear. First, effectiveness is when specified goals are achieved, such as being able to walk to a supermarket without foot pain. Secondly, efficiency is related to the relevant resources needed to achieve their goals and may include mental or physical effort of putting on and taking off shoes. The third factor is satisfaction which is related to comfort and acceptability of use. This can be considered in terms of attitudes to using the footwear for example the impact of the appearance of the footwear in the context of use such as in social environments. These factors could be used for the focus of outcome measures in practice and as Janninck et al. [32] highlighted, are essential in capturing the range of influences on usability and hence positive outcomes in both the clinical and research context.

### Barriers to measuring outcomes

Managing people with chronic disease was seen as a challenge by these participants, particularly in relation to outcomes. They discussed how goals rarely appeared to remain static during treatment and that the changes could result from the fluctuating nature of a chronic diseases. This has also been highlighted as a challenge in an orthotics service review [6].

The participants considered that measurement of outcomes for those with chronic disease created further challenges due to the multi-disciplinary interventions required. This made it difficult to identify the outcomes associated with orthoses alone. For example, therapeutic footwear has been advocated as a way of protecting the ‘at risk’ diabetic foot from ulceration [7]. However, other interventions such as regular podiatry will also contribute to this reduction of risk. Nancarrow et al. [27] expressed how in order to measure outcomes first you have to be able to define the intervention and attribute the changes in health to that specific intervention. In multi-disciplinary care where interventions can work alongside each other these specific changes can be difficult to define and attribute. To provide specificity a targeted objective clinical measure may be needed, such as the use of dynamic in shoe plantar pressure measurement to evaluate the clinical change associated with therapeutic footwear [33]. However, this does not consider the patient’s perspective on health benefits. Indeed, despite the potential for a positive outcome using a clinical measure, the patient may not find the footwear acceptable.

The participants referred to lack of training in the use of outcome measures as a barrier and this aligns with the work by Young et al. [12]. Previous studies [34–37] within other areas of allied health also found that training and education contributed to routine use of outcome measures in practice. Indeed, Jette et al. [38] highlighted that services have the responsibility to actively help clinicians manage time so that outcome measurement becomes routine.

A major barrier for these participants was the time to measure outcomes and this finding aligns with the survey of orthotists by Young et al. [12]. The issue of lack of time to administer and analyse the results has also been identified in other health professions [38–40]. Studies into the work-related experiences of orthotists and prosthetists revealed that they had insufficient time to complete their essential tasks without the addition of further, albeit desirable, activities and those with large caseloads were less likely to use outcome measures [41].

Given the importance of measuring outcomes for the various stakeholders, sufficient time is needed to ensure that this is carried out in order to drive improved and personalised care. Further, the purpose and measurement of outcomes could be included in undergraduate and post graduate training.

### Overcoming barriers to measuring outcomes

Inevitably, measuring outcomes will take up clinical time. This could result in a lower number of patients seen and result in increased waiting times for appointments [4]. However, the participants in this study expressed that measuring outcomes was an essential part of practice. In relation to overcoming lack of time as a barrier, they suggested that this task could be delegated to administrative staff, or alternatively it could be carried out in ‘snapshots’ of time, or for specific patient groups. Indeed, having administrative support has also been identified by Ross [42] as essential. It is clear is that additional time is needed to enable outcome measurement to occur and this concurs with opinions of the participants in this study.

The outcome measurement tools [43, 44] discussed within this study were found to be time consuming, and difficult to collect and analyse. Despite IT systems being considered an important instrument in minimising the burden of outcome data by aiding its selection and organisation [36], these participants expressed that they did not have access to effective IT systems. An Orthotic Pathfinder Report [3] recommendations for improvement of IT systems but it appears that there has been little improvement since 2004.

Any implementation of technology must include factors of usability [31] whilst providing accurate and meaningful data [32]. The more recent emphasis on digital health and health informatics [45] could facilitate or support the measurement of outcomes. Wider technological advances have brought wearable systems into healthcare to allow monitoring of medical conditions [46]. This could offer further options to directly assessing functional outcomes [45]. It is thought these technologies can facilitate data collection without creating or adding to clinical burden and this can reduce barriers to their use.

The study has achieved its aims, however there are potential limitations that need to be highlighted. The researcher (NH) facilitating the focus group was an orthotist could have influenced the participant’s responses during the focus groups. However, the researcher ensure that she was critical reflexive in order to enhance credibility [14, 15, 17]. It could also be said that the participants talked more freely as the researcher was the same profession. Also, this study could be challenged as having a small sample of those involved in orthotic services and hence the findings cannot be generalisable. However, this was not the purpose from the outset as it aimed to gain in-depth personal insight and opinion from 15 purposively sampled participants who demonstrated ranging years of experience in practice and a range of roles from purely clinical to those with a mixed or managerial role. Hence the findings are considered to have reasonable representation and transferability [15, 16]. Also, the concept of data saturation was not achievable and whilst we acknowledge that this is desirable in the context of rigor, the purpose of the study was to provide an in-depth interpretation of a phenomenon, that is, these participants opinions and views on outcome measures in orthotic practice.

This study informs future research in relation to measuring outcomes in a wide range of orthotic interventions for a wide range of conditions. Future research also needs to explore what outcomes are important to the wide range of patients receiving a wide range of interventions from orthotic services as these may differ from these practitioners’ perspectives. Finally, economic outcomes could be explored, and these are often of interest at a national or managerial level. They aim to analyse direct and indirect costs of care and the impact on the health of populations. Before decisions are made on the types of outcome measurement tools to employ it is vital to establish the information to be gained, the purpose it will serve and the audience for which it is meant.

## Conclusions

This study has revealed that measuring outcomes is considered to be an important activity. In order to achieve good outcomes, it is important to address patient expectations, discuss and establish joint goals for care at the outset and inform and include patients in the decision-making process. The identified barriers to measuring outcomes can be overcome with the solutions revealed by these participants. Hence, this study has contributed to current knowledge which has relevance for clinical practice and future research.

## Supplementary information

**Additional file 1.**

## Data Availability

The datasets used and/or analysed during the current study are available from the corresponding author on reasonable request.
