# Supplementary material for: An exploratory qualitative study of health professional perspectives on clinical outcomes in UK orthotic practice
Source: J Foot Ankle Res. 2020 Jul 29;13:49. doi: 10.1186/s13047-020-00416-w (PMC7392713; doi:10.1186/s13047-020-00416-w)
Supplement: Supplementary file 1 — Additional file 1. [file 13047_2020_416_MOESM1_ESM.docx]

Topic Guide questions

- “What has been your experience of evaluating outcomes for othotic devices”
- “what sort of limitations are there to the current outcome tools available”
- “ There are many types of tools available what dictates tools that you use?”
- “what outcomes do you feel are important when evaluating an orthosis”
- “What outcome measure tools do you feel are valid for use in the field?”
- “How do you find orthotic devices meet patient expectations?”
- “what factors stops you from using outcome tools regularly?”
- “how do you feel measuring outcomes influences practice?”

To gain more depth/information:

- “What else can you say about that?”, “
- “Can you give me an example?”
- “Is there anything else you can add?”
- “Can someone build on that?
- “On a scale of 1-5 how important is this?”
- “Why?” “Is there anything else you would like to add?”
- “Can you tell me more about how you felt about…?”
- “Why do you think you feel this way?”

To gain further clarity:

- “I want to make sure I understand, can you explain more?”
- “Can you give me an example?”
- “What is the best way to summarize your point for the notes?”
- “What do you mean when you say X is [‘no good’]?”
- “What does X word mean to you?

To create discussion:

- “How do others feel about that point?”
- “Who has a different perspective on that?”
- “Can someone build on that?”
